# The role of the salience network in cognitive and affective deficits

**DOI:** 10.3389/fnhum.2023.1133367

**Published:** 2023-03-20

**Authors:** Jakub Schimmelpfennig, Jan Topczewski, Wojciech Zajkowski, Kamila Jankowiak-Siuda

**Affiliations:** ^1^Behavioral Neuroscience Lab, Institute of Psychology, SWPS University, Warsaw, Poland; ^2^Center for Brain Science, RIKEN, Wako, Japan

**Keywords:** triple network model, default mode network (DMN), the frontoparietal network (FPN), salience network (SN), cognitive dysfunctions, affective dysfunctions

## Abstract

Analysis and interpretation of studies on cognitive and affective dysregulation often draw upon the network paradigm, especially the Triple Network Model, which consists of the default mode network (DMN), the frontoparietal network (FPN), and the salience network (SN). DMN activity is primarily dominant during cognitive leisure and self-monitoring processes. The FPN peaks during task involvement and cognitive exertion. Meanwhile, the SN serves as a dynamic “switch” between the DMN and FPN, in line with salience and cognitive demand. In the cognitive and affective domains, dysfunctions involving SN activity are connected to a broad spectrum of deficits and maladaptive behavioral patterns in a variety of clinical disorders, such as depression, insomnia, narcissism, PTSD (in the case of SN hyperactivity), chronic pain, and anxiety, high degrees of neuroticism, schizophrenia, epilepsy, autism, and neurodegenerative illnesses, bipolar disorder (in the case of SN hypoactivity). We discuss behavioral and neurological data from various research domains and present an integrated perspective indicating that these conditions can be associated with a widespread disruption in predictive coding at multiple hierarchical levels. We delineate the fundamental ideas of the brain network paradigm and contrast them with the conventional modular method in the first section of this article. Following this, we outline the interaction model of the key functional brain networks and highlight recent studies coupling SN-related dysfunctions with cognitive and affective impairments.

## 1. Modern paradigms in neuroimaging studies—from modular to systemic perspectives

Understanding how the brain’s rich functionality emerges from its relatively fixed anatomical structure is one of the main challenges in neuroscience. The brain’s cognitive functions can be studied at many levels of complexity, ranging from the influence of particular genes and their interactions on behavior to the analysis of dynamic systems of interdependent structures creating intrinsic brain networks. For many years, the modular perspective of applying particular functions to specific structures has dominated cognitive science (Fodor, 1983; Barrett and Satpute, 2013), most often ascribing autonomic roles to the studied regions, treating them as independent specialized modules.

However, shortcomings of this paradigm have been noted (Fuster, 2000), and study results, like the discovery of cross-modal sensory processing modulations (Garner and Keller, 2022; McClure et al., 2022), have begun to undermine even the most basic assumptions, such as monomodality of first-order sensory poles (Cappe and Barone, 2005). The biggest questions being raised regard the apparent independence and specialization of structures. Studies making this assumption often give accurate but inconclusive results in the broader context, and the lack of an overarching model makes it difficult to draw unified conclusions. The function of the anterior insula (AI) is an apt example (Wager and Barrett, 2017). Its activity is regularly attached to a wide range of apparently unrelated processes from sensory and affective processing to higher-order cognition (Uddin et al., 2017), such as body and emotional awareness, pain (Liu et al., 2021), self-recognition and motivation (Craig, 2009), singing and music recognition (Zamorano et al., 2019), uncertainty, empathy, and risky decisions (Singer et al., 2009), visual consciousness (Salomon et al., 2018), time perception (Vicario et al., 2020), attention span (Nelson et al., 2010) and integration of internal interoceptive and external sensory signals (Chen et al., 2021), as well as homeostasis (Flynn, 1999).

The systemic perspective describes psychological functions as the result of interdependent processes driven by domain-general functional brain networks, which do not have strict spatial boundaries (Park and Friston, 2013; Uddin et al., 2019; Luo, 2021). Moreover, the decoupling of structural and functional networks is required to achieve the advanced context-sensitive integration that is typical for humans (Griffa et al., 2022).

## 2. Brain functional networks

The perspective that the human brain is organized into hierarchically modularized networks is now widely accepted (Wang et al., 2015). In contrast to the assumption of independent and functionally rigid modules similar to a set of specialized tools (Gigerenzer and Todd, 1999), functional neural networks are assessed as dynamic, elastic, and hierarchical (Gilmore et al., 2018). This is necessary in order to confront changing environmental factors and develop a wide range of context-dependent behaviors (Bressler and McIntosh, 2007; Bressler and Menon, 2010). Transitions between functional networks are a response to environmental changes (Sadaghiani and Kleinschmidt, 2013). Zerbi et al. (2019) showed that a rapid reconfiguration of the functional connectome occurred in response to a threat by the release of norepinephrine, which drastically increases global brain connectivity, primarily within the salience network.

Functional neural networks have emerged from the temporally organized coupling of activity across vastly dispersed brain regions. They are characterized by the functional interdependence of brain structures within their frameworks (Bressler and Menon, 2010). Functional networks are bounded by the anatomical structure of neural connections (Xie et al., 2021). The topology of functional networks is dependent on individual development (Shanmugan et al., 2022). Furthermore, Functional connectivity (FC) can be used to predict behavioral traits such as fluid intelligence or even personality factors (NEO-FFI; Li et al., 2022). FC is a powerful tool for exploring healthy brain organization as well as mental disorders and individual differences.

**Figure 1:**
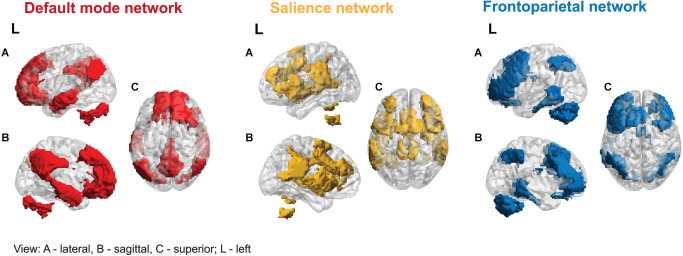
Three canonical networks.

Uddin et al. (2019) identified six prevalent macro-scale brain networks. Based on convergent evidence from many studies, three networks: Default Mode Network (DMN), Frontoparietal Network (FPN), and the Salience Network (SN), are often called canonical (Ciric et al., 2017; Uddin et al., 2022), as their interactions play a role in almost all cognitive functions (see Figure 1). The abnormal functional organization of these networks and dynamic cross-network talk may underlie a wide range of psychiatric symptoms in the “triple-network model of psychopathology” (Menon, 2018; Menon et al., 2022).

The DMN was the first large-scale network identified in human subjects and, later, across all mammalian species studied to date (Garin et al., 2022). Its central nodes consist of the posterior cingulate cortex (PCC), precuneus, and ventromedial prefrontal cortex (VMPFC; Bressler and Menon, 2010). The DMN is often referred to as a task-negative network, characterized by a stable and replicable deactivation of its core nodes during tasks requiring cognitive effort in PET and fMRI studies (e.g., Raichle et al., 2001). Nonetheless, some nodes are active throughout cognitive processing, implying that DMN plays a more complex and dynamic role in cognition (Weber et al., 2022). It was shown that the DMN is active during tasks requiring autobiographical memory, prospective thinking, ego/allocentric spatial reference, and understanding of others’ intentions (Buckner et al., 2008; Spreng et al., 2009). Additionally, the DMN is crucial for high-level social cognitive processes, mediating individual variability in cognitive empathy response (Oliveira-Silva et al., 2023).

FPN activity is significantly negatively correlated with DMN (Uddin and Menon, 2009), and its activation is relatively strongest during cognitive effort. Its function is primarily related to task selection and executive function, using input from other brain networks to actively process information, and supporting higher-order cognitive functions, such as attentional control and working memory. The FPN is also essential for decision-making in the context of goal-directed behavior in rule-based problem-solving (Lindquist and Barrett, 2012). It connects the lateral posterior parietal cortex (PPC) and the dorsolateral prefrontal cortex (DLPFC; Seeley et al., 2007).

The SN includes the AI and dorsolateral cingulate cortex (dACC; Sridharan et al., 2008). It is distinguished by a unique cellular component, the von Economo neurons in the AI/dACC (Banovac et al., 2021), characterized by a large spindle-shaped body. The SN functions as a dynamic switch between concentration on self and the inner world, mediated by the DMN, and task-related and directed attention on outside stimuli maintained by the FPN. Additionally, the amygdala and other SN subcortical nodes co-activate in response to various experimental tasks, indicating a more domain-general role in identifying homeostatically most relevant competing internal and external stimuli (Chong et al., 2017; Seeley, 2019). Its function has been shown to be relevant for processing reward, motivation, emotion, and pain (Menon, 2015).

**Figure 2:**
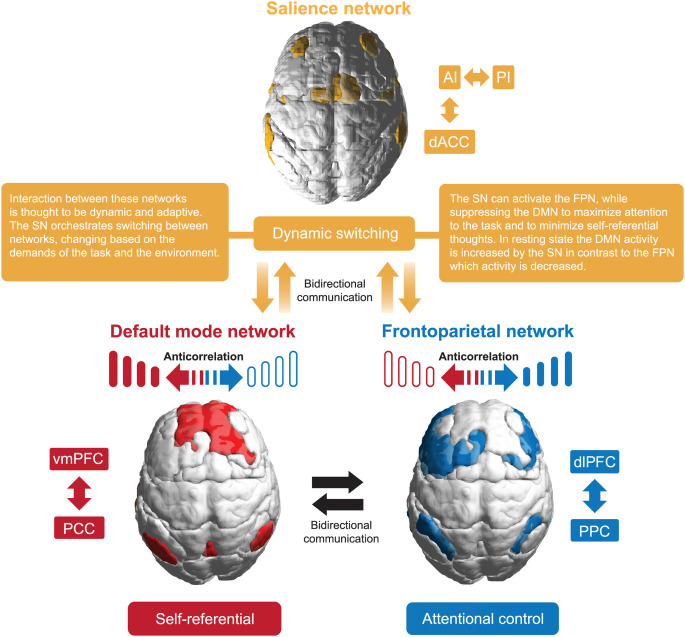
A basic interaction model of the three canonical networks.

Allocation of attentional resources to the most salient stimuli requires top-down sensitivity control and a bottom-up mechanism for filtering stimuli (Parr and Friston, 2017). A central role of the SN is filled by the insula, acting as a gatekeeper of executive control. Thanks to a widespread connectivity fingerprint, its posterior part integrates signals from within the body with external stimuli. Then, the interaction of the anterior and posterior parts of the insula moderates autonomic reactions and generates a signal sent to the anterior cingulate cortex (ACC), selectively intensifying salient stimuli that require further cortical analysis. The right dAIC is considered to be a unique brain region, functioning as a hub that influences both the FPN and DMN (Uddin, 2015). A strong negative correlation between the DMN and FPN relates to the higher efficiency of executive functions (Posner et al., 2016; see Figure 2).

## 3. Network dysfunction. The impact of network materiality on dysfunction

The correct SN response determines the appropriateness of behavior, and the AI plays a key role in the proper functioning of the entire network. Disorders within this structure are correlated with many cognitive-affective dysfunctions—including those associated with both psychiatric disorders and neurodegenerative diseases.

### 3.1. Deficits associated with an overactive salience network

Overactivity in the AI-dACC pathway is mainly associated with affective disorders (high anxiety) and neuroticism (Massullo et al., 2020). Findings indicate elevated AI activity in response to facial emotional expressions (Paulus and Stein, 2006), particularly in individuals with high levels of anxiety (Stein et al., 2007). Paulus et al. (2003) showed an association of AI with questionnaire measures—neuroticism and risk avoidance, as well as behavioral measures in making risky decisions in a gambling game. Higher activation within this structure was characteristic of riskier decisions and predicted the likelihood of choosing the safe option in the next choice. This suggests that people with elevated levels of neuroticism may interpret relatively safe situations as threatening (Feinstein et al., 2006). Hamilton et al. (2013), in a review article, presented findings showing the activity of key SN structures (AI and ACC) and the amygdala, in response to negative stimuli in depressed individuals. They also observed elevated AI activity in insomniacs when trying to fall asleep (Chen et al., 2014) and in the right AI among narcissistic individuals (Fan et al., 2011).

In addition, resting-state studies also contribute to our understanding of SN function and dysfunction. Seeley et al. (2007) noted a positive correlation between reported levels of pre-test anxiety and a measure of the strength of functional connections between AI and dACC. Markett et al. (2013), on the other hand, showed a correlation between Cloninger’s temperamental harm avoidance scale and the strength of connections between AI and ACC and AI and DLPFC. Stronger functional connectivity between dorsal ACC and new cortex regions has been reported in patients diagnosed with panic anxiety (Pannekoek et al., 2013).

It is noteworthy that SN hyperactivity is linked not only to psychological but also physical vulnerability. For example, the volume of gray matter within the insula and ACC, among others, is characteristically high in patients suffering from chronic pain (Borsook et al., 2013; Cauda et al., 2014), and the subjectively perceived level of pain is correlated with the strength of AI and ACC activations (Legrain et al., 2011).

All of the above results seem consistent with the SN model and suggest that AI overactivation leads to excessive sensitivity and anxiety arousal, and maybe a joint transdiagnostic characteristic in numerous conditions. This is likely related to the low excitability threshold of the structures that make up the SN (particularly the right AI) and the classification of excessive stimuli as important. This leads to the generalization and over-mobilization of stress reactions in non-threatening situations (Menon and Uddin, 2010; Hermans et al., 2014). In the case of narcissism, on the other hand, a proposed model of SN dysfunction relies on the inability of a dysregulated right AI to turn off the DMN, leading to an excessive concentration of thoughts on one’s self (Jankowiak-Siuda and Zajkowski, 2013).

Functional MRI data suggests that at least three subdivisions can be recognized within the insula on the basis of differential FC patterns: a dorsal anterior (dAI) involved in high-level cognitive control processes (activated by tasks requiring attention and redirecting information to the DLPFC-PPC loop), a ventral anterior (vAI) involved in affective processes (responsible for the flow of affective stimuli to specialized areas within the limbic cortex and medial prefrontal cortex), and a posterior insula (PI) involved in sensorimotor processing (Deen et al., 2011; Chang et al., 2013). Subjects with stronger connections in the ventral stream were characterized by stronger affective feelings, and subjects with stronger connections in the dorsal stream performed faster and more effectively on a cognitive task requiring the activity of attentional processes.

Alterations within all brain networks activity and connectivity in the Triple Network Model (TNM) may underlie Post Traumatic Stress Disorder (PTSD; Lebois et al., 2022). It is proposed that overactive and hyperconnected SN destabilizes intrinsically weakly connected and hypoactive DMN and FPN. In pursuance of this model, alternations in networks e.g., increased posterior SN connectivity to the PI may result in raised sensitivity to stimuli and potential threats, that contribute to avoidance and hypervigilance which characterize PTSD patients. Hyperactivation in the AI is linked with re-experiencing traumatic memories (Nicholson et al., 2020).

The SN with a low threshold for perceived saliency is not able to efficiently regulate the DMN and FPN switching (Weng et al., 2019). The impairment of cognitive control over salience processing in PTSD may be reflected in the reduced insular functional connectivity in the ACC and the supplementary motor region (Lee et al., 2022). The FPN and the DMN are weakly interconnected and hypoactive, which causes narrowed cognition and incapacity for top-down SN regulation in the FPN as well as dissociation and fear generalization in the DMN.

As shown by Fenster et al. (2018), low involvement in AI is linked to depersonalization and emotional detachment symptoms in PTSD. However, Akiki et al. (2017) suggest that alterations within the DMN may also underlie impairments in the processing of self-referential information. In addition, hyperconnectivity of the DMN with prefrontal FPN areas may limit the capacity of the FPN to engage in other cognitively demanding tasks, thus underpinning symptoms of reduced cognitive efficacy in the PTSD group. Charquero-Ballester et al. (2022) demonstrated positive correlations between activity of SN and severity of PTSD symptoms and showed that successful Cognitive Therapy for PTSD can normalize the dynamics of brain networks. The Triple Network Model offers a valuable way of comprehending the underlying neural mechanisms of PTSD, but it is unlikely to account for all PTSD abnormalities.

### 3.2. Deficits associated with underperformance of the salience network

Reduced strength of causal influence from the AI to the FPN and DMN has been linked to cognitive and affective deficits. Up until now, the best-documented links relate to schizophrenia, autism, and bipolar disorder.

Schizophrenia is characterized by impaired thinking and perception as well as shallow, maladaptive affect, which can be considered a defect of executive control. As shown by Limongi et al. (2020), the key SN nodes’ excitation-inhibition balance is impacted by the pathophysiology of glutamate neurotransmission. Reduced FC has been demonstrated between the SN and DMN (Buckner et al., 2009; Orliac et al., 2013) and between the SN and FPN (Moran et al., 2013), as well as within SN—between the AI and dACC (White et al., 2010). Structural MRI studies in people with schizophrenia have revealed a smaller volume of gray matter, encompassing all three networks (Palaniyappan et al., 2011; Krishnadas et al., 2014). The most recent research on individuals with schizophrenia revealed a general decrease in insula FC, as well as a reduction in the differentiation of connectivity profiles between insular subregions, which was associated with clinical symptom variability (Tian et al., 2019).

Orliac et al. (2013) noted negative moderate correlations between left striatum connectivity (included in the SN) and levels of hallucinations and depression. The researchers interpret this as a potential confirmation of the “relevance dysfunction” (aberrant salience) hypothesis in schizophrenia, proposed by Kapur (2003). It assumes that dysfunctional connections of the corticothalamic-parietal loop lead to chaotic discharges of dopaminergic neurons, disrupting the stimulus relevance selection taking place in the SN (Menon et al., 2022; Pugliese et al., 2022). On the other hand, Palaniyappan et al. (2013) highlighted the disruption between the SN and FPN. Granger causality analysis indicated a significantly reduced effect of SN on FPN activity, manifested by the inability to strongly engage executive structures and “mute” the DMN during cognitive effort.

The theory of predictive coding (PC) and Bayesian inference offers a comprehensive principle of brain function with the potential to link various levels of observation into a more unified model of schizophrenia (e.g., Adams et al., 2022 or Limongi et al., 2018). PC defines a biological scheme, where the brain can be seen as a computational organ generating predictions to infer the probable causes of the sensory signals, which can be compared with actual sensory samples (Friston, 2010). Bottom-up sensory evidence (information from the sensory milieu) ascends brain hierarchical architecture, where the lower levels of the brain receive predictive signals from higher levels of the brain, which encode prior beliefs. The accuracy of prediction is cyclically tested—when the incoming sensory input violates predictions, a prediction error (PE) is created and sent forward to update higher-level expectations (Bayesian belief updating; Friston, 2019). Agents weigh new evidence and prior knowledge according to the level of confidence placed in a prediction or PE, which determines the impact on belief updates. The insular cortex in this framework is seen as an integrator of low-level sensory PEs with interoceptive expectations, regulating emotion and affective salience (Barrett and Simmons, 2015).

In addition, the SN plays an essential role in the bidirectional circulation of prior beliefs, in order to execute functional integration and activation of task stimuli (Limongi et al., 2020). Royer et al. (2020) showed an insula microstructural gradient transition with changes in local affiliation: from the granular posterior, through ventral, up to agranular dorsal anterior subregions. The shift in gradient corresponded with an FC transition from primarily sensorimotor (unimodal) to modulatory and association (transmodal) networks, analogous to the hierarchical organization of other subcortical systems responsible for perceptual, control, and higher-level cognitive functions. Therefore, the multidimensional cytoarchitecture of the insular cortex (and the whole SN) is well suited for computing and transmitting the accuracy of ascending sensory PEs. The FC hierarchical gradient is considered to be a large-scale neural architecture for the PC and allostasis—predictive regulation of the body’s energy resources, which is vital for every aspect of a living organism (Katsumi et al., 2022).

The view that brain inference systems are changed in schizophrenia is supported by well-documented deficits in cognitive decision-making in numerous studies (e.g., Schmack et al., 2015; Kirihara et al., 2020). Failures of inference can explain a wide range of psychotic symptoms and traits (Friston et al., 2016). Neurotransmitter alterations underlie imprecision in the PC hierarchical mechanism, particularly in the post-synaptic gain of cortical NMDA receptors and GABAergic neurons with elevated dopaminergic neuromodulation. Disturbed neural PE signals induce misattribution of the salience of stimuli. The participation of the insula in monitoring the disruption of predictions is compatible with its function in processing salient stimuli and neuropathology in the assignment of behavioral salience to non-target stimuli in schizophrenia (Sridharan et al., 2008).

Furthermore, Luo et al. (2020) showed that control signals from rAI are improperly elevated and directed towards both the FPN and DMN, disrupting the contextually congruent assignment of brain resources in patients with schizophrenia. Liddle et al. (2016) used magnetoencephalography (MEG) to measure beta oscillations in the insula during a saliency modulation task to compare activity during task-relevant and task-irrelevant stimulus processing. Beta oscillations were chosen as they mediate endogenous long-range integrative signals or prior expectations to recurring environmental stimuli. When compared to healthy controls, schizophrenia patients had more beta synchronization in the insula when processing irrelevant stimuli over relevant ones (stronger reaction to disruption of prediction; Fries, 2015).

Empirical studies also link schizophrenia symptoms to abnormal signaling of PEs (particularly in the brain areas of reward, value-based decision-making), lack of long-term stability of internal models and priors (Sterzer et al., 2019). A DCM study of the PC provided further evidence of abnormal connectivity in the neuropathology and pathophysiology of schizophrenia (Fogelson et al., 2014). The long-standing unpredictability about upcoming sensory inputs finally leads to stimulus avoidance and psychomotor poverty, which is observed in clinical conditions (Corlett et al., 2016). In conclusion, the symptoms of schizophrenia are consistent with a decrease in high-level precision or a failure of sensory attenuation (an overestimate of the trustworthiness of the PEs), leading to false inferences and failure in cognitive control as well as the possibility of hallucinations and delusions (Sterzer et al., 2018).

Autism spectrum disorder (ASD) belongs to a group of developmental disorders characterized by qualitative abnormalities in social interactions and behavioral patterns, as well as a limited and repetitive repertoire of interests and activities (ICD-10). A meta-analysis of fMRI studies found that AI and ACC are regularly less active in people with autism, compared to a control group, during social tasks (Di Martino et al., 2009). Uddin and Menon’s (2009) model of dysfunction in autism posits that the disorder is caused by deficits in communication between sensory and limbic structures and the insula. This leads to the SN’s “underestimation” of the importance of social stimuli, which explains the phenotype of characteristic dysfunctions in responding to social stimuli. Moreover, changes in the FC pattern among the dAIC, DMN, and FPN correlate with the severity of ASD symptoms (Uddin et al., 2015). Gonzalez-Gadea et al. (2015), using the PC framework, implied that persons with ASD may have reduced precision adjustment when confronted with uncertainty because of rigid expectations (Van de Cruys et al., 2014). The predisposition to suppress bottom-up inputs and the attentional bias toward anticipated stimuli may hinder the ability to adjust precision in dynamic real-world contexts. This result is consistent with previous research on predictive coding in ASD (Lawson et al., 2014), which indicates that autistic persons struggle to contextualize sensory input in light of their preexisting beliefs and that these deficits primarily manifest in situations of uncertainty (Gomot and Wicker, 2012).

Meta-analysis of bipolar disorder (BD) patients focused on rs-fMRI and analysis of effective connectivity have shown that functional integration within and among three core brain networks (SN, DMN, and FPN) is abnormal (Sha et al., 2019; Yoon et al., 2021; Zhang et al., 2022). Altered connectivity patterns were dependent on mood, as well as the type of BD (Zhang et al., 2022). BD patients expressed altered connectivity both within networks (FPN, SN) and between (DMN-SN, DMN-FPN). There were also differences between stages of the disorder: compared to the *depression* stage, patients with *euthymic* stage expressed a hyperconnectivity among the FPN and reduced connectivity between SN and FPN and SN and DMN (Zhang et al., 2022). Martino and Magioncalda (2022) and Magioncalda and Martino (2022) suggested that the lack of integration between SN, DMN, and FPN may be due to changes in neurotransmitter signaling which can be observed during the manic and depressive phases of BD.

## 4. Discussion, limitations, and directions for further research

This mini-review segregates SN dysfunctions into hyperactivity and hypoactivity, which can lead to a simplistic perception of the mechanisms of the described deficits. However, it should be noted that the actual role of the SN in the presented disorders is more elusive. First, SN dysfunctions are a unifying feature of a whole range of deficits, but this does not mean that they are the only or even the main cause. Second, the relationships between and within SN structures themselves are complex and varied, which is one reason why the dysfunctions themselves are different. Third, atypical connections or activations within SNs are not sufficient conditions for cognitive-affective dysfunction to occur.

The second point, which entails exploring the more intricate interactions and conditional dependencies that distinguish the mechanisms underlying various disorders, seems most intriguing from the standpoint of future research. Attempts have been made to specify these mechanisms, such as the briefly described neural models of dysfunction in autism (Uddin and Menon, 2009), narcissism (Jankowiak-Siuda and Zajkowski, 2013) or schizophrenia (Kapur, 2003; Palaniyappan and Liddle, 2012). However, most of them are not yet supported by enough empirical evidence to fully validate all the hypotheses they pose; for now, they mainly serve to steer further research.

It must also be taken into account that the functional connectivity data derived from imaging studies suffer from limited spatial and temporal resolution, limiting the inference to sufficiently large brain areas and sufficiently slow dynamical processes. Coupling these findings with methods capturing millisecond dynamics (such as single or multi-electrode arrays; Spira and Hai, 2013) could lead to new insights and fuller understanding of the processes governing the network dynamics.

## Author contributions

JS: visualization, writing—original draft, writing—review and editing. JT: writing—original draft. WZ: conceptualization, writing—original draft, writing—review and editing. KJ-S: conceptualization, funding acquisition, project administration, writing—original draft, writing—review and editing. All authors contributed to the article and approved the submitted version.

## Funding

This research was partially supported by a Grant from SWPS University of Social Sciences and Humanities SUB/IPsy/04/2021/04.

## Conflict of interest

The remaining authors declare that the research was conducted in the absence of any commercial or financial relationships that could be construed as a potential conflict of interest.

## Publisher’s note

All claims expressed in this article are solely those of the authors and do not necessarily represent those of their affiliated organizations, or those of the publisher, the editors and the reviewers. Any product that may be evaluated in this article, or claim that may be made by its manufacturer, is not guaranteed or endorsed by the publisher.
